# Microbiological Profile of Pathogens Among Patients at a Tertiary Care Hospital in Mumbai, India

**DOI:** 10.7759/cureus.116551

**Published:** 2026-09-19

**Authors:** Gauravi Ranade, Pooja D Gumaste

**Affiliations:** 1 Internal Medicine, Bhatia Hospital, Mumbai, IND; 2 Microbiology, Bhatia Hospital, Mumbai, IND

**Keywords:** antibiogram, antimicrobial resistance, antimicrobial stewardship, clsi, esbl, escherichia coli, klebsiella pneumoniae, mrsa, multidrug resistant organisms

## Abstract

Background: Antimicrobial resistance (AMR) has emerged as one of the most significant threats to global public health, compromising the effective management of infectious diseases. Institutional antibiograms provide valuable information on local pathogen distribution and antimicrobial susceptibility patterns, thereby supporting evidence-based empirical therapy and antimicrobial stewardship. This study aimed to determine the microbiological profile of pathogens and their antimicrobial susceptibility patterns among patients attending a hospital in India.

Methods: A retrospective observational study was conducted in the Department of Microbiology, Bhatia Hospital, Mumbai, Maharashtra, India. Laboratory records of culture-positive clinical specimens received between January and December 2025 were reviewed. A total of 2,075 non-duplicate, clinically significant isolates obtained from various clinical specimens were included. Bacterial and fungal isolates were identified using standard microbiological methods and the VITEK® 2 Compact system (bioMérieux, Marcy-l'Étoile, France). Antimicrobial susceptibility testing was performed using the VITEK® 2 Compact system and interpreted according to the Clinical and Laboratory Standards Institute (CLSI M100, 35th edition 2025) guidelines. Organism distribution, specimen-wise and area-wise frequency, antimicrobial susceptibility profiles, and multidrug-resistant organism (MDRO) prevalence were analysed.

Results: A total of 2,075 clinically significant isolates were recovered during the study period. Gram-negative bacteria predominated, accounting for 1,627 (78.4%) isolates, followed by Gram-positive bacteria (299; 14.4%) and fungal isolates (149; 7.2%). The most frequently isolated pathogens were *Escherichia coli* (543; 26.2%), *Klebsiella pneumoniae* (519; 25.0%), *Pseudomonas aeruginosa* (328; 15.8%), *Staphylococcus aureus* (132; 6.4%), *Candida albicans* (86; 4.1%), and *Acinetobacter baumannii* complex (74; 3.6%). Midstream urine, sputum, wound swabs, catheter urine, pus swabs, tissue specimens, and blood samples constituted the major sources of isolates. MDROs accounted for 597 (28.8%) isolates, extended-spectrum β-lactamase (ESBL) producers were 369 (17.8%), methicillin-resistant *S. aureus* (MRSA) were 102 (4.9% of all isolates; 77.3% of *S. aureus* isolates), and vancomycin-resistant *Enterococcus* (VRE) accounted for 17 (0.8% of all isolates; 22.1% of *Enterococcus* isolates) with significantly higher prevalence of MDROs among isolates from critical care areas. Overall antimicrobial susceptibility testing demonstrated high resistance to several commonly used β-lactam antibiotics and fluoroquinolones, whereas reserve antimicrobial agents, including polymyxins, tigecycline, linezolid, glycopeptides, fosfomycin, and newer β-lactam/β-lactamase inhibitor combinations, retained comparatively better in vitro activity against susceptible isolates.

Conclusion: Gram-negative bacteria were the predominant pathogens isolated with a substantial burden of MDROs. The observed antimicrobial resistance patterns emphasize the need for continuous microbiological surveillance, regular updating of institutional antibiograms, and implementation of robust antimicrobial stewardship programs to optimize empirical antimicrobial therapy and improve patient outcomes.

## Introduction

Infectious diseases continue to be a major cause of morbidity and mortality worldwide and remain a substantial challenge to healthcare systems, particularly in developing countries such as India [1,2]. Hospitalized patients, especially those admitted to tertiary care centers, are at increased risk of acquiring infections caused by a wide range of bacterial and fungal pathogens. These infections contribute significantly to prolonged hospitalization, increased healthcare costs, and adverse clinical outcomes [3].

Microbiological surveillance is essential for identifying the prevalent pathogens responsible for infections and for monitoring trends in antimicrobial resistance (AMR) [4]. The spectrum of microorganisms isolated from clinical specimens varies across geographical regions and healthcare settings due to differences in patient populations, antimicrobial prescribing practices, infection control measures, and local epidemiology [5]. Therefore, periodic evaluation of the microbiological profile of pathogens is crucial for guiding empirical antimicrobial therapy and developing institutional antibiotic policies [6].

AMR has emerged as a major global public health concern [7,8]. The increasing prevalence of multidrug-resistant organisms (MDROs) [9,10], including extended-spectrum β-lactamase (ESBL), methicillin-resistant *Staphylococcus aureus* (MRSA), carbapenem-resistant gram-negative bacilli, and vancomycin-resistant enterococci (VRE), has significantly limited available treatment options [7]. The irrational use of antibiotics in both hospital and community settings has accelerated the emergence and dissemination of resistant pathogens [7].

India bears a considerable burden of AMR because of high antibiotic consumption, population density, and increasing healthcare-associated infections [1,6]. Tertiary care hospitals in metropolitan cities often manage critically ill patients with complex infections, making them important sites for studying pathogen distribution and resistance trends. Understanding the local microbiological landscape is necessary for strengthening antimicrobial stewardship and infection prevention strategies [11].

The present study was undertaken to determine the microbiological profile and antimicrobial susceptibility pattern of clinically significant bacterial and fungal isolates among patients in a hospital in Mumbai, India. The study also aimed to: (i) determine the prevalence of MDROs, including ESBL-producing *Enterobacterales*, MRSA, and VRE, along with their antimicrobial susceptibility patterns; (ii) analyse the sample-wise (specimen-wise) and area-wise distribution of pathogens detected, along with their resistance patterns; and (iii) compare the prevalence of MDROs across different clinical areas, including outpatient, general ward, and critical care settings.

## Materials and methods

This was a retrospective observational study conducted in the Department of Microbiology, Bhatia Hospital, Mumbai, Maharashtra, India. The study aimed to analyze the microbiological profile of pathogens isolated from clinically significant specimens received from both inpatient and outpatient departments. Clinical microbiology laboratory records from January 2025 to December 2025 were reviewed for the study. The study was approved by the Bhatia Hospital Medical Research Society Ethics Committee (approval number: MRS01042026).

Sample collection and processing

A total of 2,075 culture-positive clinical specimens were included in the study. Clinical samples comprised blood, urine, sputum, wound swabs, pus, body fluids, catheter tips, and other specimens submitted to the microbiology laboratory as part of routine patient care. The clinical area from which each specimen was collected included the outpatient department (OPD), general ward, intensive care unit (ICU), and burns intensive care unit; this was recorded from laboratory requisition forms for each isolate.

All specimens were collected using standard aseptic techniques and transported promptly to the microbiology laboratory for processing. Samples with incomplete laboratory data, duplicate isolates from the same patient showing identical antimicrobial susceptibility patterns, and contaminated specimens were excluded from analysis. Clinical significance was determined by the reporting microbiologist based on specimen type, quantity of growth, and correlation with the patient's clinical presentation, following standard laboratory protocols.

Isolation and identification of microorganisms

Clinical specimens were processed according to standard microbiological procedures. Samples were inoculated onto appropriate culture media, including blood agar, MacConkey agar, chocolate agar, and Sabouraud dextrose agar, depending on specimen type and suspected pathogen.

Culture plates were incubated under appropriate atmospheric and temperature conditions. Bacterial and fungal isolates were identified based on colony morphology, Gram staining, and automated identification using the VITEK 2 Compact system (bioMérieux, Marcy-l'Étoile, France). Only clinically significant isolates were included in the final analysis.

Antimicrobial susceptibility testing

Antimicrobial susceptibility testing was performed using the VITEK 2 Compact system (bioMérieux). Minimum inhibitory concentration (MIC)-based susceptibility results were interpreted as susceptible, intermediate, or resistant according to current Clinical and Laboratory Standards Institute guidelines (CLSI M100, 35th edition 2025) [12].

Antibiotics tested included β-lactams, cephalosporins, carbapenems, aminoglycosides, fluoroquinolones, glycopeptides, and other routinely used antimicrobial agents based on organism-specific testing panels. As the antimicrobial panels tested differed according to organism group and CLSI M100, 35th edition (2025) recommendations, not all isolates were tested against every antimicrobial agent; the number of isolates tested for each antibiotic therefore reflects only those isolates actually tested against that agent, not the total study cohort (n = 2,075).

Detection of MDROs

MDROs were defined as isolates showing non-susceptibility to at least one antimicrobial agent in three or more antimicrobial categories, according to the standardized international criteria proposed by Magiorakos et al. [9]. ESBL production was identified using phenotypic confirmatory methods according to standard guidelines. MRSA was identified using cefoxitin resistance, while VRE were identified based on vancomycin MIC interpretation.

Data collection and statistical analysis

Microbiological and antimicrobial susceptibility data were extracted from laboratory records and entered into Microsoft Excel (Microsoft Corporation, Redmond, Washington, United States) for analysis. Categorical variables were expressed as frequencies and percentages. Associations between pathogen distribution, specimen type, clinical area of isolation, and AMR patterns were assessed using the Chi-square test or Fisher's exact test. For area-wise analysis, the intensive care unit and burns intensive care unit were combined into a single 'critical care' category and compared against general ward and outpatient department specimens; isolates lacking documented area of collection (n=7) were excluded from the area-wise analysis. A p-value of <0.05 was considered statistically significant. Statistical Analyses were performed using GraphPad Prism version 10 (Dotmatics, Boston, Massachusetts, United States).

## Results

Overall microbiological profile

A total of 2,075 non-duplicate, culture-positive clinical isolates were recovered during the study period from patients attending Bhatia Hospital. Gram-negative bacteria constituted the majority of isolates (n=1,627; 78.4%), followed by Gram-positive bacteria (n=299; 14.4%) and fungal isolates (n=149; 7.2%).

Distribution of clinical specimens

Culture-positive isolates were obtained from a wide variety of clinical specimens. Midstream urine constituted the largest proportion of specimens (n=600; 28.9%), followed by sputum (n=338; 16.3%), wound swabs (n=233; 11.2%), catheter urine (n=198; 9.5%), pus swabs (n=129; 6.2%), pus (n=119; 5.7%), tissue specimens (n=95; 4.6%), blood (n=85; 4.1%), endotracheal secretions (n=43; 2.1%), bronchoalveolar lavage (BAL) (n=34; 1.6%), transtracheal secretions (n=25; 1.2%), other body fluids (n=22; 1.1%), bile (n=15; 0.7%), tracheal aspirates (n=13; 0.6%), and drain fluid (n=12; 0.6%). The remaining isolates were recovered from other clinical specimens submitted to the microbiology laboratory.

Distribution of major pathogens

Among Gram-negative bacteria,* Escherichia coli* and *Klebsiella pneumoniae* were the predominant pathogens, accounting for 543 and 519 isolates, respectively, followed by *Pseudomonas aeruginosa* (328 isolates) and *Acinetobacter baumannii* complex (74 isolates). Among Gram-positive bacteria, *S. aureus *was the most frequently isolated organism (132 isolates). Fungal isolates were predominantly represented by *Candida* species (86 isolates).

Sample-wise and area-wise distribution of pathogens and their resistance profile

Analysis of the five most frequently isolated pathogens across major specimen categories revealed distinct site-specific patterns linked to differing resistance burdens. *E. coli* was predominantly urinary in origin (307 of 543 isolates, 56.5%, from midstream urine), an organism with an overall MDR rate of 22.7%. *K. pneumoniae* was predominantly respiratory (184 of 519 isolates, 35.5%, from sputum), with an overall multi-drug resistance (MDR) rate of 54.1%. *P. aeruginosa* was distributed across sputum (78/328, 23.8%), wound swabs (62, 18.9%), and midstream urine (56, 17.1%), with an overall MDR rate of 43.6%. *S. aureus *was predominantly recovered from suppurative and wound specimens (pus and pus swabs combined, 52/132, 39.4%; wound swabs, 29, 22.0%), with 77.3% of isolates being MRSA (Table 1).

**Table 1:** Sample-wise distribution of dominant organisms MDR: multidrug resistance; MRSA: methicillin-resistant *Staphylococcus aureus*

Specimen	Dominant organism	Percentage of the organism from the specimen	Organism's overall resistance rate
Urine (Midstream)	Escherichia coli	307/543 = 56.5%	MDR 22.7%
Sputum	Klebsiella pneumoniae	184/519 = 35.5%	MDR 54.1%
Sputum	Pseudomonas aeruginosa	78/328 = 23.8%	MDR 43.6%
Wound swab	Pseudomonas aeruginosa	62/328 = 18.9%	MDR 43.6%
Wound swab	Staphylococcus aureus	29/132 = 22.0%	MRSA 77.3%
Pus + Pus swab (combined)	Staphylococcus aureus	52/132 = 39.4%	MRSA 77.3%
Urine (Catheter)	Escherichia coli	54/543 = 9.9%	MDR 22.7%
Urine (Catheter)	Klebsiella pneumoniae	51/519 = 9.8%	MDR 54.1%
Tissue	Pseudomonas aeruginosa	23/328 = 7.0%	MDR 43.6%

Admission area was documented for 2,068 of 2,075 isolates (99.7%); the remaining seven isolates (all *Candida* species) lacked area-of-collection data and were excluded from area-wise analysis. MDRO prevalence differed significantly by clinical area, rising from 26.2% (208/793) in outpatient specimens and 25.2% (197/781) in general wards to 38.9% (192/494) in critical care areas (intensive care unit and burns intensive care unit combined).

MDROs

Of the 2,075 isolates, 597 (28.8%) were identified as MDROs. Among the major Gram-negative pathogens, 281 isolates of *K. pneumoniae*, 123 isolates of *E. coli*, 143 isolates of *P. aeruginosa*, and 46 isolates of *A. baumannii* complex were classified as multidrug-resistant. The remaining four multidrug-resistant isolates were distributed among *Serratia marcescens* (n=2), *Enterobacter cloacae* complex (n=1), and *Proteus mirabilis* (n=1).

**Figure 1:**
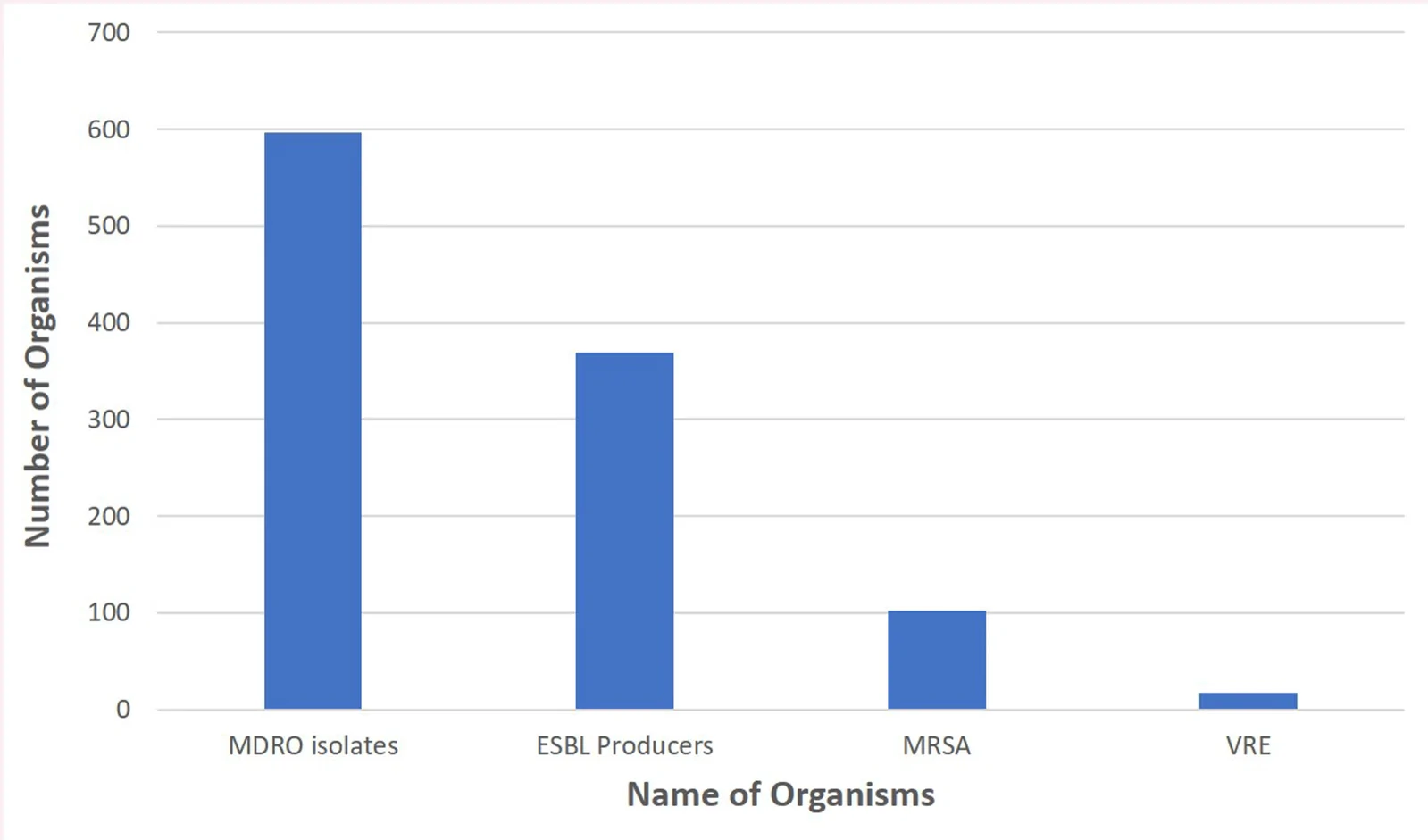
Distribution of resistant organisms MDRO: multidrug-resistant organisms; ESBL: extended-spectrum β-lactamase; MRSA: methicillin-resistant *Staphylococcus* *aureus; *VRE*:  *vancomycin-resistant *Enterococcus *

ESBL-producing organisms accounted for 369 (17.8%) isolates. MRSA was detected in 102 (4.9%) isolates, whereas methicillin-susceptible *S. aureus* (MSSA) accounted for 30 (1.4%) isolates. VRE was identified in 17 (0.8%) isolates (Figure 1). Organism-specific resistance patterns are described in Table 2.

**Table 2:** Comparison of organism-specific and whole-cohort resistance rates MRSA: methicillin-resistant *Staphylococcus aureus*; VRE: vancomycin-resistant *Enterococcus; *ESBL: extended spectrum β-Lactamase

Resistance phenotype	Number of isolates	Organism-specific denominator	Organism-specific percentage	Percentage of total isolates (n=2,075)
MRSA	102	132 (*Staphylococcus aureus*)	77.30%	4.90%
VRE	17	77 (*Enterococcus* spp.)	22.10%	0.80%
ESBL — *Escherichia coli*	253	543	46.60%	12.20%
ESBL — *Klebsiella pneumoniae*	102	519	19.70%	4.90%

Antimicrobial susceptibility profile

Antimicrobial susceptibility testing revealed considerable variation in the activity of commonly used antimicrobial agents. The number of isolates tested varied by agent, reflecting organism-specific testing panels rather than the total isolate count. Among β-lactam/β-lactamase inhibitor combinations, amoxicillin-clavulanate demonstrated a susceptibility of 26.8% (69/257 isolates), while ampicillin-sulbactam showed 26.2% susceptibility (67/256 isolates). Piperacillin-tazobactam demonstrated 25.8% susceptibility (60/233 isolates).

Marked resistance was observed against several penicillins and cephalosporins. Ticarcillin exhibited the highest resistance rate, with 94.8% (602/635) of tested isolates resistant. Resistance to ticarcillin-clavulanate reached 98.4% (121/123), while penicillin resistance was 98.1% (204/208).

Among cephalosporins, cefuroxime demonstrated 21.5% susceptibility (326/1,515 isolates), cefixime 24.3% (261/1,076), and cefotaxime 24.0% (382/1,589). These findings indicate widespread resistance to third-generation cephalosporins among the isolates included in the study.

The overall antibiogram demonstrated extensive resistance to several routinely prescribed β-lactam agents, emphasizing the limited utility of these antibiotics for empirical therapy in the study setting. In contrast, reserve antimicrobial agents-including carbapenems, polymyxins, tigecycline, glycopeptides, oxazolidinones, and newer β-lactam/β-lactamase inhibitor combinations-showed comparatively better in vitro activity and remain important therapeutic options for multidrug-resistant infections. Detailed organism-wise susceptibility patterns are presented in the corresponding antibiogram tables.

Statistical analysis of associations

Associations between pathogen distribution, specimen type, admission setting, and AMR were assessed using the chi-square test and Fisher’s exact test. MDRO rates differed significantly across the four major Gram-negative pathogens (χ² = 127.58, df = 3, p<0.0001), with *A. baumannii *complex (62.2%) and *K. pneumoniae* (54.1%) showing the highest proportions of MDR, compared with *P. aeruginosa* (43.6%) and *E. coli* (22.7%). Organism distribution varied significantly across specimen types (χ² = 597.5, df = 28, p<0.0001). ESBL positivity was significantly higher among *E. coli* (46.6%) than *K. pneumoniae* (19.7%) isolates (Fisher’s exact test, OR = 3.57, p<0.0001). Susceptibility to meropenem (χ² = 131.6, df = 3, p<0.0001) and piperacillin-tazobactam (χ² = 53.4, df = 3, p<0.0001) differed significantly among the four major Gram-negative organisms, with *K. pneumoniae* and *A. baumannii *complex showing the lowest susceptibility. MDRO prevalence differed significantly across clinical areas of isolation (χ² = 31.79, df = 2, p<0.0001). This area-related difference was organism-dependent: resistance prevalence rose significantly with increasing care intensity for *P. aeruginosa* (χ² = 13.81, df = 2, p = 0.001), *A. baumannii* complex (χ² = 11.77, df = 2, p = 0.0028), and MRSA positivity among *S. aureus* isolates (χ² = 7.78, df = 2, p = 0.020), whereas *E. coli* (p = 0.305) and *K. pneumoniae* (p = 0.066) showed no statistically significant area-related difference. A p-value <0.05 was considered statistically significant for all comparisons.

## Discussion

The present study provides a comprehensive overview of the microbiological profile and antimicrobial susceptibility patterns of clinically significant pathogens isolated from patients attending Bhatia Hospital, Mumbai, India, over a one-year period. Analysis of 2,075 non-duplicate, culture-positive isolates demonstrated a predominance of Gram-negative bacteria (78.4%), followed by Gram-positive bacteria (14.4%) and fungal isolates (7.2%). These findings are consistent with reports from tertiary care hospitals across India, where Gram-negative bacilli continue to account for the majority of healthcare-associated and community-acquired bacterial infections [1,6].

Among the isolated pathogens, *E. coli *(26.2%) and *K. pneumoniae *(25.0%) were the most frequently recovered organisms, together accounting for approximately half of all isolates. *P. aeruginosa* (15.8%) and *A. baumannii* complex (3.6%) were also important contributors, particularly in respiratory, wound, and intensive care unit specimens, consistent with their recognized status as WHO critical-priority pathogens for AMR surveillance [13]. Notably, *Candida albicans* (4.1%) was the most frequently isolated fungal species. The predominance of these organisms reflects the increasing burden of Gram-negative bacterial infections in tertiary healthcare settings and highlights the need for continuous microbiological surveillance [8].

The distribution of specimens demonstrated that midstream urine, sputum, wound swabs, catheter urine, and pus swabs constituted the major sources of culture-positive isolates. This pattern is comparable with previous hospital-based studies, where urinary tract infections, lower respiratory tract infections, and wound infections represent the most common indications for microbiological culture [5]. The diversity of specimen types included in the present study improves the representativeness of the institutional antibiogram and enhances its utility in guiding empirical antimicrobial therapy across different clinical specialties. Notably, respiratory and wound specimens were dominated by organisms carrying substantially higher resistance rates (*K. pneumoniae*, 54.1% MDR; *P. aeruginosa*, 43.6% MDR; *S. aureus*, 77.3% MRSA) compared with the predominantly urinary *E. coli* (22.7% MDR).

A major finding of the present study was the high prevalence of MDROs (28.8%). ESBL-producing organisms constituted 17.8% of all isolates, indicating continued dissemination of ESBL-mediated resistance among *Enterobacterales* [14]. The high number of multidrug-resistant *K. pneumoniae* and *E. coli *isolates is of particular concern because these pathogens are among the leading causes of urinary tract infections, bloodstream infections, intra-abdominal infections, and surgical site infections [2,15]. Similar trends have been reported from several Indian surveillance studies, reflecting the widespread circulation of resistant *Enterobacterales* in hospital environments [1,6]. Comparable resistance trends among Gram-negative and Gram-positive pathogens have also been documented by regional and national surveillance networks in Europe and the United States, underscoring the global scale of the AMR challenge [16-18].

MRSA accounted for 102 isolates, whereas VRE represented a smaller proportion of isolates. Although glycopeptide resistance remained relatively uncommon, the presence of MRSA and VRE emphasizes the importance of maintaining robust infection prevention measures, routine screening where appropriate, and strict adherence to antimicrobial stewardship programs to limit transmission within healthcare facilities [18,19].

The overall antibiogram, a tool increasingly recognized as central to guiding local antimicrobial policy [20], demonstrated extensive resistance to several routinely prescribed β-lactam antibiotics and fluoroquinolones. Reduced susceptibility to third-generation cephalosporins is consistent with the observed prevalence of ESBL-producing organisms. In contrast, reserve antimicrobial agents, including polymyxins, tigecycline, linezolid, vancomycin, teicoplanin, daptomycin, fosfomycin, and ceftazidime-avibactam, retained comparatively better in vitro activity against susceptible isolates [21]. These findings support current antimicrobial stewardship recommendations that reserve these agents for infections caused by multidrug-resistant pathogens in order to preserve their clinical effectiveness [22].

The predominance of multidrug-resistant *P. aeruginosa* and *A. baumannii* complex further highlights the challenges associated with managing healthcare-associated infections, particularly in critically ill patients [12,23]. These organisms possess multiple intrinsic and acquired resistance mechanisms, limiting available therapeutic options and increasing the risk of prolonged hospitalization, higher treatment costs, and adverse clinical outcomes [3]. Consistent with this, resistance prevalence rose significantly with increasing care intensity for *P. aeruginosa*, *A. baumannii* complex, and MRSA positivity among *S. aureus *isolates, whereas *E. coli *and *K. pneumoniae* showed no significant area-related difference, likely reflecting the cumulative effect of prolonged hospital stay, invasive device use, prior antibiotic exposure, and greater patient acuity in critical care settings. Continuous monitoring of resistance trends is therefore essential for updating institutional antibiotic policies [24].

The findings of the present study have important implications for hospital infection control and antimicrobial stewardship, supporting regular updating of institutional antibiograms to enable early detection of emerging resistance trends [5,25].

The strengths of this study include the large sample size, inclusion of a broad range of clinical specimens, area-wise analysis of pathogens, standardized organism identification and antimicrobial susceptibility testing using an automated platform, and interpretation according to the CLSI M100, 35th edition (2025) guidelines [12]. The inclusion of MDRO surveillance further enhances the clinical applicability of the study findings for antimicrobial stewardship programs.

Nevertheless, certain limitations should be acknowledged. This was a retrospective, single-center study, and therefore the findings may not be directly generalizable to other healthcare settings. Molecular characterization of resistance mechanisms, including carbapenemase and ESBL genes, was not performed. In addition, patient-level clinical variables, antibiotic exposure history, and treatment outcomes were beyond the scope of the present analysis. Future multicenter prospective studies incorporating molecular epidemiology and clinical outcome assessment would provide a more comprehensive understanding of AMR patterns and their impact on patient care.

## Conclusions

The present study provides a comprehensive overview of the microbiological profile and antimicrobial susceptibility patterns of clinically significant pathogens isolated from patients attending a hospital in India. Gram-negative bacteria were the predominant pathogens, with *E. coli*, *K. pneumoniae*, and *P. aeruginosa* accounting for the majority of isolates. A considerable proportion of isolates were identified as MDROs, including ESBL-producing *Enterobacterales*, MRSA, and VRE, highlighting the increasing burden of AMR in the hospital setting. Notably, resistance was not uniformly distributed across the hospital: MDRO prevalence was higher among isolates from critical care areas compared with general wards and outpatient settings, driven particularly by *P. aeruginosa*, *A. baumannii* complex, and MRSA. The overall antibiogram demonstrated reduced susceptibility to several commonly prescribed antibiotics, particularly β-lactams and fluoroquinolones, whereas reserve antimicrobial agents retained comparatively better in vitro activity against susceptible isolates.

These findings emphasize the importance of regular microbiological surveillance, updated institutional antibiograms, and robust infection prevention and stewardship programmes to contain the emergence and spread of AMR. The findings of this study provide valuable baseline data for clinicians, microbiologists, infection control teams, and hospital administrators to strengthen antimicrobial stewardship strategies and optimize the management of infectious diseases in tertiary healthcare settings.
